# The use of neutrophil elastase inhibitor in the treatment of acute lung injury after pneumonectomy

**DOI:** 10.1186/1749-8090-8-69

**Published:** 2013-04-08

**Authors:** Sang Kwon Lee, Bong Soo Son, Jung Joo Hwang, Kil Dong Kim, Do Hyung Kim

**Affiliations:** 1 Department of Thoracic and Cardiovascular Surgery, Pusan National University Yangsan Hospital, Yangsan, South Korea; 2 Department of Thoracic and Cardiovascular Surgery, Eulji University Hospital, Daejeon, South Korea

## Abstract

**Background:**

The prognosis of acute lung injury (ALI) after pneumonectomy is poor, with reported mortality rates of 30-100%. Neutrophil elastase inhibitor (NEI) is known to prevent lung injury caused by neutrophil elastase and improve lung function in ALI. We evaluated the effect of NEI on ALI after pneumonectomy.

**Methods:**

We analyzed nine patients who required ventilator care due to ALI after pneumonectomy. Five of these patients underwent conventional ventilator care (group I), and four patients underwent ventilator care and were administrated NEI (group II). We retrospectively analyzed the lung injury score (LIS) for 10 days after intubation.

**Results:**

The LIS before intubation satisfied the diagnostic criteria of ALI or acute respiratory distress syndrome (ARDS) in all patients. After intubation, the LIS improved in both groups. But, as times went on, the mean value of the LIS in group II was lower compared to group I. In group I, only one patient underwent extubation. In group II, extubation was possible in three patients. Mortality rates were 80% in group I and 25% in group II.

**Conclusions:**

We conclude that NEI may improve the lung function, shorten the duration of mechanical ventilation, and reduce mortality in patients with ALI after pneumonectomy.

## Background

Acute lung injury (ALI) after lung resection may be due to systemic inflammatory reactions syndrome or lymphatic drainage dysfunction [1]. The incidence of ALI after pneumonectomy is 2–5 times higher than the incidence after lobectomy [2]. Furthermore, the prognosis of ALI after pneumonectomy is considerably poor, with reported mortality rates of 30-100% [3]. Therefore, a substantial amount of effort has been focused on increasing the survival rate of ALI after pneumonectomy [4].

Recently, several studies have reported that sivelestat, a new neutrophil elastase inhibitor (NEI) drug, prevents lung injury caused by neutrophil elastase and improves the lung function in ALI [5,6]. Sivelestat is also known to shorten the duration of mechanical ventilation and reduce mortality of ALI. However, there are only a few clinical reports investigating whether NEI has positive effects on ALI after pneumonectomy. In this study, we evaluate the effects of sivelestat on ALI after pneumonectomy.

## Methods

From April 2004 to December 2010, nine patients required ventilator care due to ALI after pneumonectomy. We retrospectively reviewed the medical records. Table 1 shows the characteristics of the patients. We used sivelestat (Elaspol®, ONO Pharmaceutical Co., Osaka, Japan) in the treatment of acute lung injury or acute respiratory failure since September 2006. We classified two groups according to using of suvekestat. Five of these patients underwent conventional ventilator care only, and these were defined as group I. Four patients received ventilator care and were administered sivelestat. This patient group was defined as group II. Sivelestat was administrated immediately after intubation and was continuously infused at rate of 0.2 mg/kg/h for 10 days. There was no difference of treatment policy except sivelestat between two groups.

**Table 1:** Patient characteristics

**Number**	**Sex**	**Age**	**Cause**	**Pneumonectomy side**	**NEI**	**Extubation**	**Survival**	**Onset of ALI (POD)**
1	Male	45	Pneumonia	Right	No	Yes	Yes	4
2	Male	55	Aspiration	Right	No	No	No	147
3	Male	42	Pneumonia	Right	No	Yes*	No	204
4	Male	67	Pneumonia	Right	No	No	No	91
5	Male	77	Pulmonary edema	Left	No	No	No	1
6	Male	66	Pneumonia	Left	Yes	Yes	Yes	36
7	Male	63	Pneumonia	Left	Yes	Yes	Yes	9
8	Male	68	Pneumonia	Left	Yes	No	No	26
9	Male	54	Aspiration	Right	Yes	Yes	Yes	6

*Reintubation after extubation.

NEI, neutrophil elastase inhibitor; ALI, acute lung injury; POD, postoperative day.

We used Murray’s acute lung injury score (LIS) to assess the extent of lung injury [7]. This scoring system has four components: chest X-ray score, hypoxemia score, positive end expiratory pressure (PEEP) score, and respiratory system compliance score (Table 2). The final value is obtained by dividing the aggregate sum by the number of components that were used. We calculated the LIS at the time of pre-intubation, post-intubation, and every day for 10 days following post-intubation. When the final value of the LIS was less than 0.5, patients were weaned off the ventilator. All values are reported as mean ± standard deviation. Prior to data collection, we obtained approval from the ethics committee of Eulji University Hospital. Informed consent was obtained from the patient or families for publication.

**Table 2:** Lung injury score (Murray score)

1. Chest roentgenogram score		
No alveolar consolidation		0
Alveolar consolidation confined to 1 quadrant		1
Alveolar consolidation confined to 2 quadrant		2
Alveolar consolidation confined to 3 quadrant		3
Alveolar consolidation in all 4 quadrant		4
2. Hypoxemia score		
PaO_2_/FiO_2_	≥300	0
PaO_2_/FiO_2_	225-299	1
PaO_2_/FiO_2_	175-224	2
PaO_2_/FiO_2_	100-174	3
PaO_2_/FiO_2_	<100	4
3.PEEP score (when ventilated)		
PEEP	≤5 cm H_2_O	0
PEEP	6-8 cm H_2_O	1
PEEP	9-11 cm H_2_O	2
PEEP	12-14 cm H_2_O	3
PEEP	≥15 cm H_2_O	4
4. Respiratory system compliance score (when available)		
Compliance	≥80 ml/cmH_2_O	0
Compliance	60-79 ml/cmH_2_O	1
Compliance	40-59 ml/cmH_2_O	2
Compliance	20-39 ml/cmH_2_O	3
Compliance	≤19 ml/cmH_2_O	4
	Score	
No lung injury	0	
Mild to moderate lung injury	0.1-2.5	
Severe lung injury (ARDS)	>2.5	

PaO_2_/FiO_2_, arterial oxygen tension to inspired oxygen concentration ratio; PEEP, positive end-expiratory pressure; ARDS, acute respiratory distress syndrome.

## Results

The LIS before intubation was 3.0±0.0 in group I and 3.0±0.0 in group II. These values before intubation satisfied the diagnostic criteria of ALI or acute respiratory distress syndrome (ARDS) in all patients. The mean value of the LIS after intubation and mechanical ventilation rapidly decreased to 2.3±0.2 (range = 2.0-2.7) in group I and 2.5±0.1 (range = 2.3-2.7) in group II. The mean value of the LIS on post-intubation day 1 further improved to 1.5±0.7 (range = 0.7-2.3) in group I and 1.4±0.6 (range = 0.7-2.0) in group II. Improvements in the LIS appeared in both groups regardless of the use of sivelestat.

**Figure 1:**
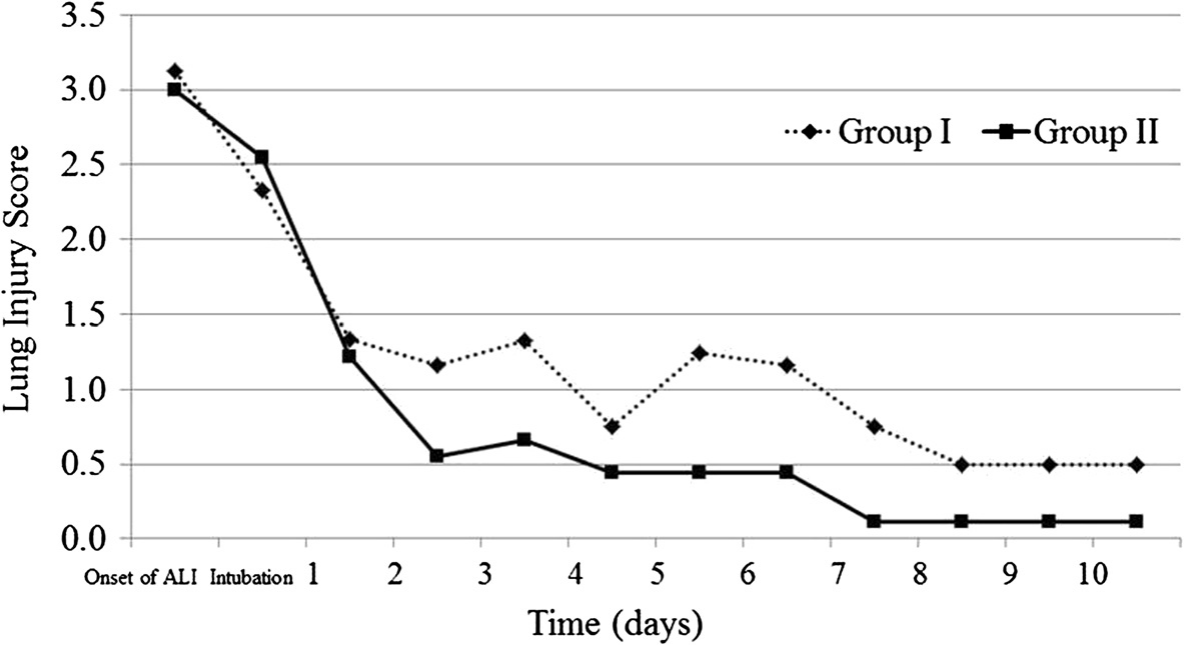
Changes in lung injury score from the onset of ALI to 10 days after intubation.

The mean value of LIS in group II continued to decrease with time when compared with the LIS of group I (Figure 1). In group I, only two patients (40%) achieved an LIS less than 0.5, the score consider low enough to wean the patient off the mechanical ventilator. These two patients achieved this score after 7 and 10 days of ventilator care, respectively. Only one of these two patients was able to undergo extubation. The other patient required re-intubation due to immediate deterioration of respiratory function after extubation, and he could not be weaned off the ventilator thereafter. In contrast to group 1, in group II three patients (75%) achieved an LIS of less than 0.5 within 7 days after intubation and were successfully weaned off the ventilator. Only one patient in this group (who suffer from an acute myocardial infarction) did not achieve an LIS of 0.5 or less.

All patients who failed in being weaned off the ventilator died. The overall mortality rate was 80% in group I and 25% in group II. All deaths (n=4) in group I were related to respiratory failure. In group II, the only death was due to acute myocardial infarction. This patient’s value of arterial oxygen tension to inspired oxygen concentration ratio (PaO_2_/FiO_2_) was 302.2 mmHg.

## Discussion

ALI is caused by neutrophils which are activated by systemic inflammatory reactions. Activated neutrophils migrate to the lung and secrete elastase which induces the secondary migration of neutrophils. This process damages vascular endothelial or pulmonary epithelial cells, which accelerates the lung damage [8].

Several animal studies have reported that NEI can inhibit the elastase-dependent migration of neutrophils and the production of cytokines [9,10]. Therefore, NEI is expected to attenuate the progression of ALI [11]. NEI has already been used clinically for treating ALI [12].

However, it still remains controversial whether NEI has positive effects on all types of ALI. Zeiher et al. [13] reported that NEI did not significantly affect mortality rate or ventilator free day. Bernard et al. [14] also reported that NEI did not significantly alter the mortality of ARDS.

Because patients who have undergone pneumonectomy have lower pulmonary function, ALI after pneumonectomy seems to show specific pattern of clinical progress when compared to different types of ALI. First, patients with ALI after pneumonectomy generally have severe respiratory symptoms, with a PaO_2_/FiO_2_ less than 100 at the time of diagnosis. Second, the symptoms occur early in comparison with the real degree of ALI progression. Third, the values of PaO2/FiO2 are recovered immediately after mechanical ventilation. We see this in the patients in our study. These patients had a mean value of more than 250 mmHg on post-intubation day 2.

Considering the pharmacokinetics of NEI, NEI should be used in the early stage of ALI to maximize the curative effects. Hoshi et al. [6] reported that the use of NEI in the early stage of ALI could shorten the duration of mechanical ventilation and reduce mortality. However, in cases of severe ALI where many neutrophils have already migrated to the lung and induced alveolar damages, NEI was unable to significantly improve symptoms. The symptoms of ALI after pneumonectomy are severe. However, such severe symptoms may help provide an early diagnosis and proper course of treatment. Therefore, administering NEI along with mechanical ventilation upon observed declined lung functions in ALI after pneumonectomy has allowed for the rapid recovery of lung function.

It generally takes a long time to fully recover lung function up to the pre-ALI level, and prolonged mechanical ventilation causes ventilator-induced secondary lung injuries. Since NEI is able to shorten the duration of ventilation, we speculate it could also reduce the incidence of ventilator-induced lung injury which may reduce the incidence of complications in pneumonectomy patients.

## Conclusions

In summary, patients who were administered NEI along with mechanical ventilation after pneumonectomy had a lower LIS, a shorter duration of ventilation, and a higher success rate of ventilator weaning compared to patients who received only ventilator therapy. Treatment with NEI also resulted in lower overall mortality. This study has important limitations including a small subject number and retrospective analysis. However, our data indicate that NEI treatment leads to improved the lung function in early ALI patients and warrants further investigation.

## Abbreviations

ALI: Acute lung injury; NEI: Neutrophil elastase inhibitor.

## Competing interests

The authors declare that they have no competing interests.

## Authors’ contributions

SKL, JJH and DHK participated in the design of the study. BSS, and KDK collected data. SKL, JJH and DHK reviewed literature and wrote the article. All authors read and approved the final manuscript.
